# Emotions, preventive measures, and risk perception in post-emergency COVID-19: a cross-sectional study in Italian university staff during the seasonal vaccination campaign

**DOI:** 10.1186/s12889-026-26782-x

**Published:** 2026-03-14

**Authors:** Michela Sarlo, Giuditta Fiorella Schiavano, Chiara Orlandi, Giorgio Brandi, Anna Casabianca

**Affiliations:** 1 Department of Communication Sciences, Humanities and International Studies, University of Urbino Carlo Bo, Via Saffi 15, Urbino, 61029 Italy; 2 Department of Humanities, University of Urbino Carlo Bo, Urbino, Italy; 3 Department of Biomolecular Sciences, University of Urbino Carlo Bo, Urbino, Italy; 4 Covid-Lab, University of Urbino Carlo Bo, Fano, Italy

**Keywords:** COVID-19, Post-emergency, COVID-19 vaccination campaign, Risk perception, Antigen testing, Emotions.

## Abstract

**Background:**

Despite the end of the global health emergency, COVID-19 is expected to persist and continue to pose a threat to public health, due to the emergence of new virus variants and the risks of long-term effects. Therefore, seasonal vaccinations for vulnerable populations (i.e., older adults and individuals with underlying medical conditions) and extensive testing to detect infection remain crucial strategies for protecting public health. This study aimed to investigate attitudes, intentions, behaviors, and emotions related to COVID-19, vaccination, and the use of antigen tests among university staff during the 2023–2024 COVID-19 vaccination campaign in Italy.

**Method:**

An ad hoc anonymous survey was administered to 267 university staff members, aged 18–70 years, between February 15 and March 20, 2024. Logistic regression models were estimated to evaluate the determinants of vaccination adherence, risk perception, and test-taking intention.

**Results:**

Adherence to the vaccination campaign was low (13.1% overall; 30.6% among individuals aged ≥ 60) and was positively associated with older age (OR = 1.84, CI [1.01, 3.33]) and with a greater number of previous vaccine doses (OR = 22.65, CI [7.00, 73.28]). Trust/hope toward vaccination increased the likelihood of adherence (OR = 1.76, CI [1.10, 2.82]), whereas higher early-pandemic anxiety/worry reduced it (OR = 0.46, CI [0.27, 0.77]). Individuals who had never contracted COVID-19 were less likely to perceive high infection risk (OR = 0.31, CI [0.17, 0.58]), and higher early-pandemic anxiety/worry was associated with greater perceived risk (OR = 1.31, CI [1.06, 1.62]); however, risk perception did not predict COVID-19 vaccination adherence or test-taking intentions. The intention to take an antigen test was greater among individuals who adopted more preventive measures, such as mask use, distancing, and hygiene behaviors (OR = 1.56, CI [1.19, 2.05]), who had received more vaccine doses (OR = 2.24, CI [1.22, 4.11]), and who reported greater trust/hope toward testing (OR = 2.18, CI [1.46, 3.27]).

**Conclusions:**

Overall, the findings underscore the central role of emotional responses and past preventive behaviors, rather than risk perception, in shaping both COVID-19 vaccination adherence and test-taking intentions during the post-emergency phase.

**Supplementary Information:**

The online version contains supplementary material available at 10.1186/s12889-026-26782-x.

## Introduction

On May 5, 2023, the World Health Organization (WHO) [1] declared the end of the global COVID-19 health emergency. In Italy, the removal of restrictions (e.g., mandatory isolation for positive cases, mask requirements), together with the marked reduction of COVID-related information through mass media and institutional channels, has rapidly shaped preventive behaviors, risk perception, and attitudes toward the disease. However, COVID-19 is expected to persist and may continue to threaten global health due to new emerging variants and the possibility of long-term debilitating effects (long COVID) across age groups and disease severity [2]. The need for ongoing monitoring and surveillance, as well as for ensuring vaccination in vulnerable populations – namely, older adults and individuals with underlying medical conditions (e.g., cardiovascular disease, diabetes, chronic respiratory disease, or cancer) – is therefore reaffirmed [1]. In particular, routine or seasonal vaccinations may be required to address declining immunity and protect against highly transmissible or immune-evading variants [3]. In parallel, extensive testing remains crucial for detecting symptomatic and asymptomatic infections, with rapid antigen self-tests offering an accessible alternative to laboratory-based diagnostics and enabling individual contributions to public health [4].

Between December 1, 2023, and January 31, 2024, a total of 287,532 new cases of contagion and 2,583 deaths were reported in Italy, with a peak in mid-December (60,440 new cases, 425 deaths, over 7,500 hospitalizations, and more than 260 intensive-care admissions) [5]. Notably, according to the European Centre for Disease Prevention and Control (ECDC) [6], Italy was one of the countries with the lowest COVID-19 vaccination coverage during the 2023–2024 season campaign, with only 6% of individuals aged 60–69, 11.7% of those aged 70–79, and 15.8% of those aged ≥ 80 years receiving the vaccine. This occurred despite the Italian Ministry of Health circular (September 27, 2023), which defined the targets of the 2023–2024 COVID-19 vaccination campaign and recommended vaccination for individuals aged ≥ 60 years and/or with severe vulnerabilities, as well as for their family members and caregivers [7].

The failure of the 2023–2024 COVID-19 vaccination campaign can be attributed to multiple factors beyond logistical and organizational challenges (e.g., delays in vaccine distribution, and limited engagement of general practitioners and pharmacies). In particular, recent research has increasingly acknowledged the negative impact of ‘pandemic fatigue’ on information-seeking motivation [8], vaccine compliance [9], self-health monitoring [10], and the adoption of preventive measures [11]. Pandemic fatigue refers to the physical, cognitive and emotional burden posed by pandemic restrictions, policies, and recommendations [12]. While still under debate [13], this emerging concept highlights the significant role of higher emotional distress, lower risk perception, and weaker trust in government and public health authorities in reducing tendencies to adhere to health-protective behaviors [14]. Moreover, news media coverage on COVID-19 plays a major role in shaping risk perception while reflecting society’s current concerns [15].

According to the influential account of Slovic and colleagues [16], risk perception is strongly influenced by automatic and intuitive emotional processes, which shape risk judgments based on experience and the positive or negative feelings elicited by the situation (*risk as feeling*). On the other hand, judgments driven by affective processes can be regulated and adjusted by cognitive reflective processes that rely on logic and statistical reasoning (*risk as analysis*), provided that the decision-maker has sufficient time and information [17]. Empirical research consistently indicates that risk perception plays a crucial role in vaccination intention [18, 19] and in the adoption of protective measures [20, 21]. Furthermore, perceived vaccine safety has been shown to be a strong predictor of vaccination intention [22, 23]. However, the way individuals perceive risk and respond to health recommendations is strongly shaped by the social and organizational contexts in which they operate [24]. Understanding how individuals working within structured institutional environments perceive COVID-19-related risks, recommendations, and preventive measures is particularly relevant in the post-emergency phase, as these groups were repeatedly exposed to official communication, organizational constraints, and workplace health policies during the pandemic. Within this perspective, university staff represent a particularly informative study population. Unlike more heterogeneous general populations, individuals working within academic institutions experienced prolonged organizational constraints and common workplace regulations throughout the pandemic, thereby providing a shared contextual background in which to examine post-emergency attitudes and behaviors.

Drawing from existing research, we considered three broad domains of determinants that have been identified as crucial for COVID-19 protective decision-making: cognitive-evaluative factors, informational exposure and trust, and affective responses. These domains have been shown to shape health-protective decisions and guided the selection of the constructs examined. Within this framework, pandemic fatigue might provide relevant contextual background for interpreting post-emergency attitudes, including the current disengagement from COVID-19 vaccination, testing practices, and preventive behaviors that motivated the present research.

This study was conducted during Italy’s 2023–2024 COVID-19 vaccination campaign, and aimed to investigate attitudes, intentions, behaviors, and affective responses related to COVID-19, vaccination, and the use of antigen tests among university staff in the post-emergency phase. Specifically, we examined four areas of interest: (a) factors associated with adherence to the vaccination campaign, considering cognitive-evaluative, informational, affective, and behavioral components previously linked to protective health behaviors; (b) determinants of risk perception, drawing on evidence that experiential, informational, and affective cues shape judgments of infection risk and disease severity; (c) predictors of intention to take an antigen test in case of symptoms, focusing on cognitive, informational, affective, and behavioral factors relevant to testing decisions; (d) differences in affective states across pandemic conditions (COVID-19, vaccination, antigen testing) and phases (retrospective appraisal of pandemic onset vs. current experience), given the central role of affect in COVID-19 decision-making.

Following contemporary affective science, emotional experience is understood to include both discrete emotions and broader feeling states. “Emotions” refer to short-lived and categorically defined reactions elicited by specific appraisals of threat or norm violation (e.g., fear, guilt), whereas “feeling states” denote more diffuse and sustained affective experiences shaped by ongoing appraisals and motivational tendencies (e.g., anxiety, worry, trust) [25]. In this study, the term “affective states” is used to encompass both components.

## Methods

### Study sample

The study sample consisted of 267 participants (173 F), aged 18–70 years, affiliated with the University of Urbino Carlo Bo, a medium-sized university in Central Italy. The sample included faculty members, technical-administrative staff, research fellows, and doctoral candidates. All participants belonged to the same institutional setting and had direct exposure to COVID-19-related organizational measures and institutional communication during the pandemic, ensuring a homogeneous contextual background across the sample.

Participants selected their age within predefined 10-year ranges (18–29, 30–39, 40–49, 50–59, ≥ 60 years) to preserve anonymity and minimize the risk of indirect identification, thereby ensuring that they felt comfortable reporting potentially sensitive information, including attitudes toward COVID-19 vaccination.

### Procedure

This study employed a single-center, cross-sectional design, with all variables measured at a single time point via an anonymous online survey (Google Forms) distributed to 1,110 university affiliates. All currently employed faculty members, technical-administrative staff, research fellows, and doctoral candidates with an institutional e-mail address were invited to participate via an institutional e-mail sent by the University Covid-Lab. The message briefly described the aims of the study, specified that participation was voluntary and anonymous, and contained a direct link to the survey. There were no additional inclusion or exclusion criteria beyond active institutional affiliation and age ≥ 18 years. Since the entire eligible population was approached, no a priori sample size calculation was performed.

Data collection took place between February 15 and March 20, 2024. Before accessing the questionnaire, potential participants were presented with an information page describing the study aims, procedures, data protection and confidentiality, and their right to withdraw at any time. Only those who provided electronic informed consent could proceed to the survey. The respondents were not offered any incentives for participation. The study was not preregistered.

### Measures

An ad hoc questionnaire was developed specifically for this study, drawing on recent literature on COVID-19 risk perception, vaccination attitudes, and preventive behaviors [26, 27], and on WHO guidelines for COVID-19 risk communication and vaccination campaigns [28]. The instrument was not based on previously validated multi-item scales; rather, items were created by the authors to assess key aspects of participants’ COVID-19-related experiences, adapting constructs and indicators commonly used in previous research. The full English version of the questionnaire, including all response options, is provided in the Supplementary Material (Appendix A).

The questionnaire consists of 9 thematic areas (33 items in total) designed to explore multiple aspects of the COVID-19 experience: demographic and health information; prior experience with COVID-19; access to COVID-19 information; trust in sources of COVID-19 information; risk perception; use of antigen tests in case of symptoms; vaccination against COVID-19; current preventive behavior; and the intensity of the affective states related to contracting COVID-19 (retrospectively assessed for the onset of the pandemic and in real time for the current phase), as well as those currently related to vaccination and the use of antigen tests. All thematic areas were assessed using structured items with predefined response formats, as detailed in Table 1.

**Table 1:** Study variables, response options, and variable types

Variable	Response options	Variable type
*Demographic and health information*		
Sex	Female / Male / Prefer not to answer	Categorical (nominal)
Age group	18–29 / 30–39 / 40–49 / 50–59 / ≥60 years	Categorical (ordinal)
Educational level	Primary / Lower secondary / Upper secondary / Advanced artistic, musical, and choreographic education / Bachelor’s / Master’s / PhD	Categorical (ordinal)
Cohabitation context	Living alone / With person(s) aged ≥ 60 / With person(s) with chronic illness or severe vulnerabilities/ None of the above	Categorical (nominal)
Perceived health status	Poor / Fair / Good / Excellent	Categorical (ordinal)
Chronic illness	Yes / No	Dichotomous
*Prior experience with COVID-19*		
COVID-19 infection (self)	Yes / No	Dichotomous
COVID-19 severity (self)	Asymptomatic / Mild / Moderate / Severe	Categorical (ordinal)
COVID-19 infection (family members)	Yes / No	Dichotomous
COVID-19 severity (family members)	Asymptomatic / Mild / Moderate / Severe / Deceased	Categorical (ordinal)
*Access to COVID-19 information*		
COVID-19 info accessibility	Very easy / Easy / Difficult / Very difficult	Categorical (ordinal)
COVID-19 info dissemination	Never / Rarely / Sometimes / Often / Very often	Categorical (ordinal)
Accessibility of instructions in case of infection	Very easy / Easy / Difficult / Very difficult	Categorical (ordinal)
Awareness of workplace COVID-19 regulations	Yes / No	Dichotomous
*Trust in sources of COVID-19 information*		
Applied separately to television, newspapers, healthcare professionals, social media, radio, Ministry of Health, National Institute of Health, and World Health Organization	None / Little / Moderate / Much / Complete	Likert-type (ordinal)
*Risk perception*		
Perceived likelihood of contracting COVID-19	Unlikely / Fairly likely / Very likely	Categorical (ordinal)
Perceived severity of possible illness	Asymptomatic / Mild / Moderate / Severe	Categorical (ordinal)
Perceived severity of current COVID-19 spread	Not severe at all / Slightly severe / Moderately severe / Very severe	Categorical (ordinal)
*Use of antigen tests in case of symptoms*		
Test intention	Yes / No	Dichotomous
Reasons for testing (yes)	Multiple predefined + Other	Categorical (nominal, descriptive only)
Reasons for not testing (no)	Multiple predefined + Other	Categorical (nominal, descriptive only)
*Vaccination against COVID-19*		
Trust in COVID-19 vaccine safety	None / Little / Moderate / Much / Complete	Likert-type (ordinal)
Number of vaccine doses received	Numeric entry	Continuous (count variable)
Awareness of the 2023–2024 vaccination campaign recommendations	Yes / No	Dichotomous
Adherence to the 2023–2024 vaccination campaign	Yes / No / Reserved	Categorical (nominal)
Reasons for vaccination (yes)	Multiple predefined + Other	Categorical (nominal, descriptive only)
Reasons for non-vaccination (no)	Multiple predefined + Other	Categorical (nominal, descriptive only)
Household COVID-19 vaccination status (2023–2024)	Yes / No / Not all of them / Not applicable	Categorical (nominal)
*Current preventive behavior*		
Preventive measures adopted (e.g., handwashing, surface disinfection, mask use, physical distancing)	Multiple predefined + Other	Categorical (count variable)
*Affective states*		
At the thought of contracting COVID-19 (current phase and pandemic onset), as well as of COVID-19 vaccination and the use of antigen tests (current phase)	Predefined affective states: fear, sadness, anger/frustration, anxiety/worry, stress, sense of trust/hope, sense of calm/tranquility, pride, guilt, shame, sense of helplessness, sense of uncertainty, and indifference 0 (none/no intensity) – 5 (maximum intensity)	Likert-type (ordinal)

The selection of the affective states was based on their relevance to the psychological and social dynamics of the COVID-19 pandemic. Basic negative emotions (i.e., fear, sadness, anger) are universally recognized responses to threats, losses, or challenges [29]. Anxiety/worry and stress reflect anticipatory or ongoing threat appraisals [30]. Perceived lack of control (i.e., helplessness, uncertainty) can hinder engagement in active behaviors [31]. Moral emotions (i.e., guilt, shame, pride) regulate social behavior and adherence to norms [32]. Positive feeling states (i.e., trust/hope, calm/tranquility) may buffer distress and support adaptive coping under uncertainty [33]. Finally, indifference reflects emotional disengagement or lack of concern [34].

Because the questionnaire is composed of conceptually distinct sections and is not intended to form a single unidimensional scale, global internal consistency indices were not computed.

### Data analysis

Descriptive statistics were computed for the variables of interest.

Affective states were treated as approximately continuous variables in the analyses. A repeated-measures analysis of variance (ANOVA) was conducted on the mean intensity of current affective states, with *Pandemic Condition* (COVID-19, vaccination, and antigen test) and *Affective State* (the affective states described above) as within-subjects factors. Additionally, a repeated-measures ANOVA was conducted on the mean intensity of affective states related to COVID-19, with *Pandemic Phase* (current vs. onset of pandemic) as a within-subjects factor. To control for type I errors, the Greenhouse-Geisser (G-G) correction was applied when necessary. The uncorrected degrees of freedom are reported together with the adjusted probability values. Tukey HSD post-hoc tests were employed to further examine significant effects (*p* < .05).

A binary logistic regression was conducted to examine the factors associated with *adherence to the 2023–2024 vaccination campaign* (0 = No, 1 = Yes). Independent variables included sex, age group, educational level, cohabitation context, perceived health status, severity of previous COVID-19 infection for oneself and family members, number of vaccine doses received, trust in COVID-19 vaccine safety, awareness of recommendations for the 2023–2024 vaccination campaign, perceived likelihood of contracting COVID-19, perceived severity of current COVID-19 spread, and affective states (trust/hope and anxiety/worry about getting vaccinated, anxiety/worry about contracting COVID-19 currently and at the onset of the pandemic).

Two ordinal logistic regressions were conducted to examine the factors associated with *risk perception*: the perceived likelihood of contracting COVID-19 (unlikely, fairly likely, very likely) and the perceived severity of the current COVID-19 spread in Italy (not severe at all, slightly severe, moderately severe, very severe). Independent variables included sex, age group, educational level, cohabitation context, perceived health status, personal infection history, number of vaccine doses received, adherence to the 2023–2024 vaccination campaign, awareness of recommendations for the 2023–2024 vaccination campaign, access to COVID-19 information, frequency of dissemination through mass media, awareness of current workplace COVID-19 regulations, and affective states (anxiety/worry about contracting COVID-19 currently and at the onset of the pandemic).

Finally, a binary logistic regression was conducted to examine the factors associated with the *intention to take an antigen test in case of symptoms* (0 = No, 1 = Yes). Independent variables included sex, age group, educational level, cohabitation context, perceived health status, severity of previous COVID-19 infection for oneself and family members, perceived likelihood of contracting COVID-19, perceived severity of current COVID-19 spread, number of preventive measures adopted, number of vaccine doses received, accessibility of instructions in case of infection, trust in the Ministry of Health, and affective states (trust/hope and anxiety/worry about testing, anxiety/worry about contracting COVID-19 currently and at the onset of the pandemic).

Across these models, we selected partially overlapping but not identical predictors drawn from the three theoretical domains introduced above (cognitive-evaluative, informational/trust, and affective), guided by their outcome-specific relevance and while preserving parsimony and interpretability given the sample size, particularly the limited number of vaccinated participants. Predictor selection was theoretically driven and a priori, aiming to identify conceptually and empirically justified variables for each specific outcome rather than to test a single unified predictive framework. For example, the vaccination adherence model included demographic and health-related variables (e.g., age, health status, and cohabitation with older adults or vulnerable individuals) to reflect eligibility criteria and priority targets defined by the national vaccination campaign. This analytical strategy aligns with the exploratory aim of the study, which focused on identifying distinct predictors for distinct post-emergency outcomes.

Categorical predictors were entered as factors, with one category specified as the reference group where applicable. The age group was coded into five predefined categories (18–29, 30–39, 40–49, 50–59, and ≥ 60 years). The number of previous COVID-19 vaccine doses (0–6) and the number of preventive measures adopted (0–7) were entered as numeric predictors. Affective states were treated as numeric predictors based on 6-point Likert-type scales (0–5), with higher values indicating greater intensity of the corresponding affective state.

The regression models were estimated using the enter method, and the results are reported as odds ratios (OR) with 95% confidence intervals (CIs). The logistic regressions included 266 participants, as one case was excluded due to a data entry error in the number of vaccine doses. For four variables (educational level, perceived severity of possible illness, perceived severity of current COVID-19 spread, and number of vaccine doses received), one level was collapsed with an adjacent one due to insufficient frequency (< 5 cases) to ensure statistical validity [35].

All the statistical analyses were performed using IBM SPSS Statistics (version 29.0), except for the post-hoc Tukey HSD tests following the ANOVAs, which were carried out using TIBCO^®^ Statistica (version 14.0).

## Results

### Descriptive analysis

The main characteristics of the sample are reported in Table 2.

**Table 2:** Sociodemographic characteristics, health status, COVID-19 experience, and vaccination history

		*N*	%
Sex	Male	94	35.2
	Female	173	64.8
Age group (years)	18–29	13	4.9
	30–39	49	18.3
	40–49	43	16.1
	50–59	100	37.5
	≥ 60	62	23.2
Educational level	Lower secondary school	3	1.1
	Upper secondary school	27	10.1
	Advanced artistic, musical, and choreographic education	1	0.4
	Bachelor’s degree	11	4.1
	Master’s degree	104	39.0
	PhD	121	45.3
Cohabitation context	Living alone	42	15.7
	With persons aged ≥ 60	76	28.5
	With persons with chronic illness/severe vulnerabilities	21	7.9
	None of the above	128	47.9
Perceived health status	Poor	6	2.2
	Fair	27	10.1
	Good	194	72.7
	Excellent	40	15.0
Chronic illness	No	211	79.0
	Yes	56	21.0
COVID-19 (self)	No	66	24.7
	Asymptomatic	12	4.5
	Mild	104	38.9
	Moderate	80	30.0
	Severe	5	1.9
COVID-19 (family members)	No	22	8.2
	Asymptomatic	6	2.2
	Mild	75	28.1
	Moderate	137	51.3
	Severe	14	5.2
	Deceased	13	5.0
Number of vaccine doses received	0	8	3.0
	1	3	1.1
	2	30	11.3
	3	164	61.6
	4	45	16.9
	5	14	5.3
	6	2	0.8
Awareness of the 2023–2024 COVID-19 vaccination campaign recommendations	No	116	43.4
	Yes	151	56.6

The majority of the sample (68.9%) had previously experienced a mild or moderate form of COVID-19, as had most of their family members (79.4%). Most participants (61.6%) received three vaccine doses, whereas only 16.9% received the fourth dose, despite strong recommendations. An even smaller proportion (5.3%) received the fifth dose, which included protection against the Omicron variant.

Overall, 23.2% of the participants were aged 60 years or older and, according to current recommendations, should have received the vaccination. Moreover, 36.4% lived with at least one person aged 60 years or older, or with chronic illnesses/severe vulnerabilities (Table 2), and were also recommended for vaccination. In contrast, only 13.1% of the sample adhered to the COVID-19 vaccination campaign, including only 30.6% of those aged ≥ 60 years (Table 3). Among those who received the vaccine, 77.2% reported doing so for protection against infection, regardless of their risk status. On the other hand, among those who were not vaccinated, the primary reasons were not perceiving COVID-19 as a serious health threat (31%) and feeling sufficiently protected due to prior infection (27.2%). Notably, 21.1% either questioned the vaccine’s effectiveness/safety or were concerned about its potential side effects. In this regard, a total of 22.1% of the respondents reported having little to no trust in the safety of the COVID-19 vaccine (Table 4).

**Table 3:** Distribution of vaccinated and non-vaccinated participants across different age groups in the 2023–2024 COVID-19 vaccination campaign

Age group	Yes			No		
	*N*	% Within age group	% Within vaccinated	*N*	% Within age group	% Within non-vaccinated
18–29 years	0	0.0	0.0	13	100.0	5.6
30–39 years	3	6.1	8.6	46	93.9	19.9
40–49 years	1	2.3	2.8	42	97.7	18.1
50–59 years	12	12.0	34.3	88	88.0	37.9
≥ 60 years	19	30.6	54.3	43	69.4	18.5

Percentages within age groups indicate the proportion of vaccinated or non-vaccinated individuals in each category, whereas percentages within vaccination status indicate the age distribution within the vaccinated and non-vaccinated groups

**Table 4:** Trust in COVID-19 vaccine safety and in sources of information about COVID-19

	None		Little		Moderate		Much		Complete	
	*N*	%	*N*	%	*N*	%	*N*	%	*N*	%
COVID-19 vaccine safety	21	7.9	38	14.2	72	27.0	89	33.3	47	17.6
Television	52	19.5	115	43.1	84	31.5	14	5.2	2	0.7
Newspapers	39	14.6	111	41.6	99	37.1	16	6.0	2	0.7
Radio	35	13.1	108	40.5	102	38.2	20	7.5	2	0.7
Social media	118	44.2	111	41.6	36	13.5	2	0.7	0	0.0
Healthcare professionals	7	2.6	18	6.7	72	27.0	127	47.6	43	16.1
National Institute of Health	12	4.5	23	8.6	63	23.6	108	40.5	61	22.8
Ministry of Health	15	5.6	25	9.4	78	29.2	105	39.3	44	16.5
World Health Organization	15	5.6	19	7.1	66	24.8	101	37.8	66	24.7

Pairwise comparisons were performed using Wilcoxon signed-rank tests with Bonferroni correction for multiple tests

Overall, 18.7% of the sample reported intending not to take an antigen test in case of symptoms, with this percentage rising to over 30% in those aged 18–29 years. Among participants who would not take the test, the primary reason (52%) was that they would not change their behavior or habits even if they tested positive. Among those who would take the test, the primary reason (71.4%) was to prevent transmission of the virus to others, extending beyond just family or household members.

Overall, 55.4% of the sample considered contracting COVID-19 fairly likely and, overall, 66.6% expected to experience only a mild form of the illness. Moreover, 59.9% considered the current COVID-19 spread to be slightly severe. Notably, while 51.7% adopted between 2 and 4 preventive measures (in particular, social distancing, handwashing, and avoiding crowded places), 15.4% reported no longer following any preventive measures (Table 5).

**Table 5:** Risk perception and preventive measures adopted

		*N*	%
Perceived likelihood of contracting COVID-19	unlikely	92	34.5
	fairly likely	148	55.4
	very likely	27	10.1
Perceived severity of possible illness	asymptomatic	33	12.4
	mild	178	66.6
	moderate	55	20.6
	severe	1	0.4
Perceived severity of current COVID-19 spread	not severe at all	43	16.1
	slightly severe	160	59.9
	moderately severe	62	23.3
	very severe	2	0.7
Number of preventive measures adopted	0	41	15.4
	1	50	18.7
	2	34	12.7
	3	67	25.1
	4	37	13.9
	5	22	8.2
	6	13	4.9
	7	3	1.1

With respect to access to information about COVID-19, only 56.6% of the sample were aware of the recommendations for the 2023–2024 vaccination campaign (Table 2), whereas 65.5% were aware of current workplace regulations for COVID-19 positivity.

Considering trust in sources of information about COVID-19 (Table 4), the Friedman test revealed significant differences (χ²(7) = 1197.452, *p* < .001, Kendall’s W = 0.64). Pairwise comparisons (Wilcoxon signed-rank tests) indicated that healthcare professionals, the Ministry of Health, the National Institute of Health, and the WHO received the highest level of trust (all ps < .001), with no significant differences among them (all ps > .08). Newspapers, television, and radio showed intermediate levels of trust, ranking below health authorities and above social media. Overall, social media received the lowest level of trust (all ps < .001).

### Affective states

**Fig. 1:**
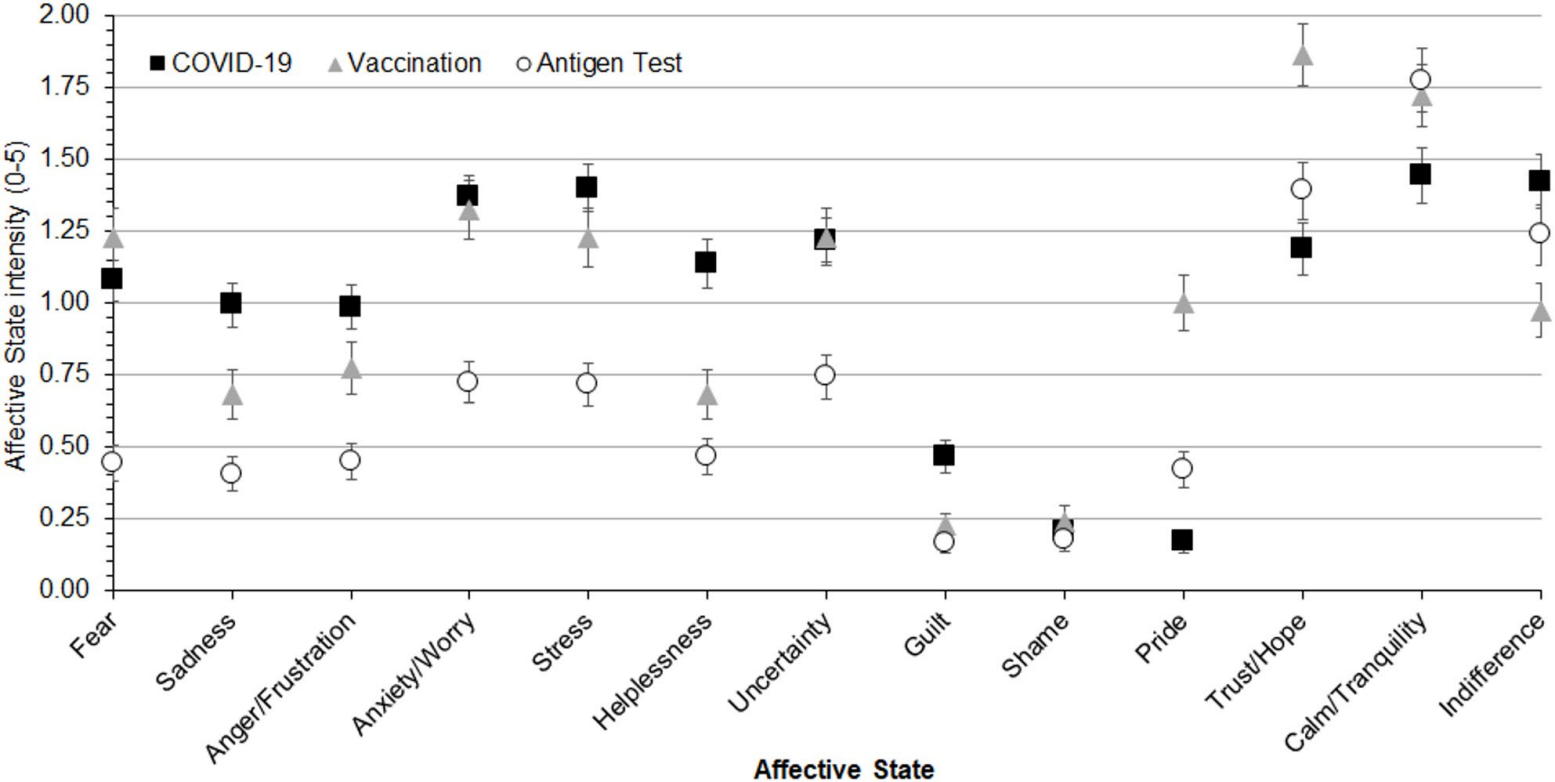
Mean intensity ratings of affective states across the three assessed pandemic conditions (COVID-19, vaccination, and antigen test). The error bars represent standard errors. Points are displayed without connecting lines to avoid implying a temporal trend

The ANOVA on the intensity of *current* affective states revealed significant main effects of *Pandemic Condition* (F[2,532] = 35.39, *p* < .0001, ε = 0.91, η^2^_p_ = 0.12) and *Affective State* (F[12,3192] = 67.97, *p* < .0001, ε = 0.31, η^2^_p_ = 0.20). Post-hoc tests indicated that, overall, the thought of undergoing an antigen test elicited significantly lower emotional intensities than contracting COVID-19 or getting vaccinated (ps < .0001). Across all conditions, calm/tranquility was the most intensely reported affective state (all ps < .0001). The *Pandemic Condition* × *Affective State* interaction was significant (F[24,6384] = 16.46, *p* < .0001, ε = 0.30, η^2^_p_ = 0.06; Fig. 1). The thought of contracting COVID-19 and receiving vaccination elicited comparable levels of fear, anxiety/worry, and stress (all ps > .99). However, COVID-19 elicited greater helplessness and indifference, whereas vaccination elicited greater trust/hope and pride (all ps < .0001). The thought of undergoing an antigen test generally elicited lower emotional intensities across most affective states. Compared with COVID-19, it elicited significantly lower levels of fear, anxiety/worry, stress, anger/frustration, sadness, helplessness, and uncertainty (all ps < .0001). Similarly, compared to vaccination, antigen testing elicited significantly lower levels of fear, anxiety/worry, stress, trust/hope, pride, and uncertainty (all ps < .0001). Examining emotional intensity within each condition, fear, anxiety/worry, stress, and helplessness were among the most intensely reported feelings in the COVID-19 condition. Calm/tranquility and indifference were also rated at comparably high levels, with no significant differences among all these affective states (all ps > 0.11). In the vaccination condition, trust/hope and calm/tranquility were the most intensely reported states (all ps < .005), whereas in the antigen test condition calm/tranquility was the most dominant affective state (all ps < .0001), followed by trust/hope.

**Fig. 2:**
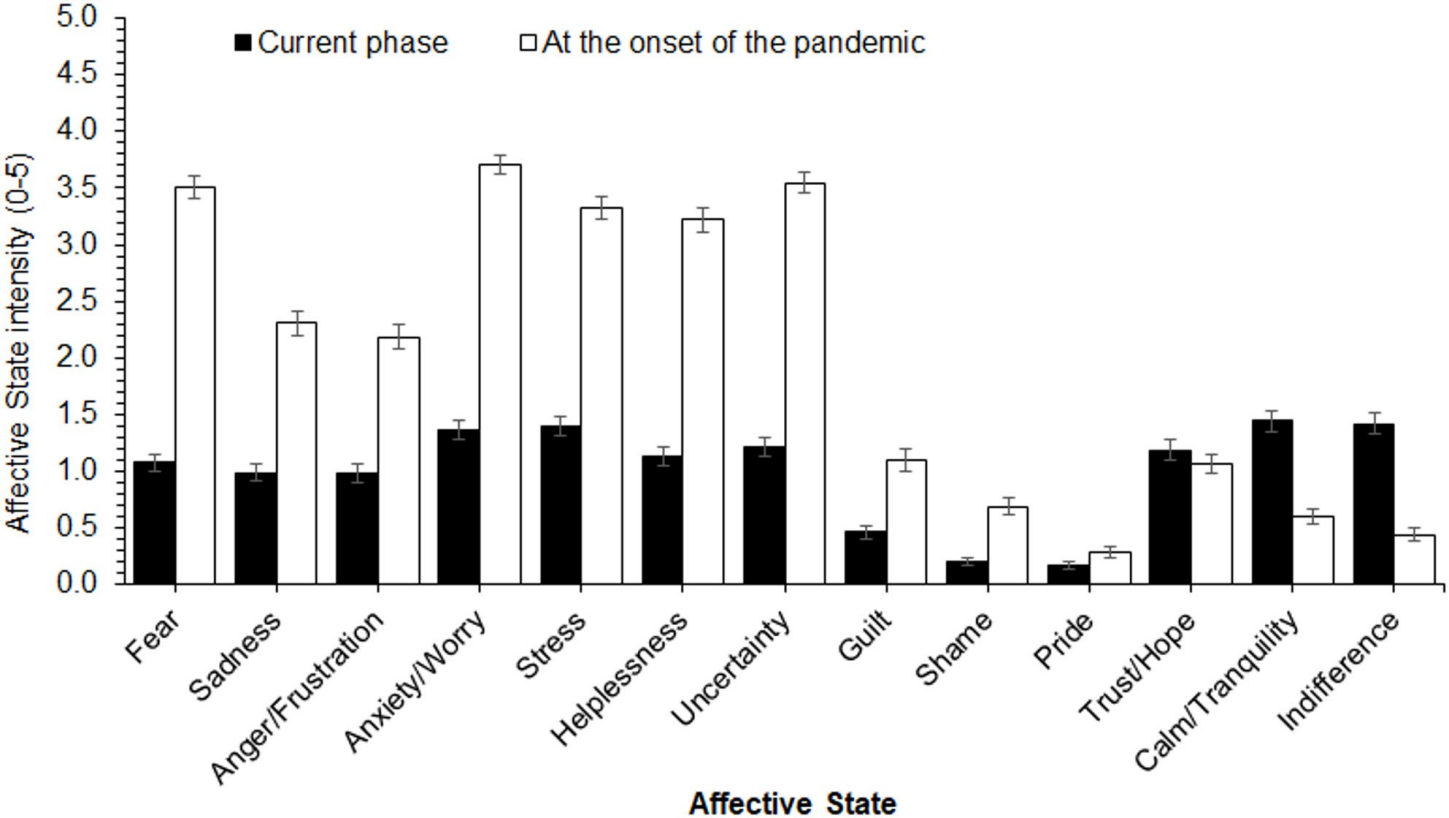
Mean intensity ratings of affective states related to COVID-19 as a function of the pandemic phase. The error bars represent standard errors of the means

The ANOVA examining differences in the intensity of affective states related to COVID-19 across pandemic phases revealed significant main effects of *Pandemic Phase* (F[1, 266] = 544.69, *p* < .0001, ε = 1.0, η^2^_p_ = 0.67) and *Affective State* (F[12,3192] = 182.70, *p* < .0001, ε = 0.37, η^2^_p_ = 0.41). These effects were qualified by the significant *Affective State* × *Pandemic Phase* interaction (F[12,3192] = 209.99, *p* < .0001, ε = 0.59, η^2^_p_ = 0.44; Fig. 2). Overall, the intensity of most affective states was markedly higher at the onset of the pandemic than in the current phase (all ps < .0001). In contrast, calm/tranquility and indifference were reported with greater intensity in the current period (ps < .0001). Only trust/hope and pride showed no significant differences across pandemic phases (ps > 0.99). Fear, anxiety/worry, stress, helplessness, and uncertainty were the affective states most intensely reported at the thought of contracting COVID-19 at the onset of the pandemic.

### Regression models

#### Adherence to the 2023–2024 COVID-19 vaccination campaign

The binary logistic regression on adherence to vaccination revealed a significant model (χ²(16) = 122.32, *p* < .0001; Nagelkerke’s R² = 0.68). Older participants (across the 18–29, 30–39, 40–49, 50–59, and ≥ 60 years age groups) had higher odds of vaccination adherence (OR = 1.84, CI [1.01, 3.33], *p* = .045). Individuals with a greater number of previous vaccine doses (ranging from 0 to 6) had markedly higher odds of vaccination adherence (OR = 22.65, CI [7.00, 73.28], *p* < .001). Individuals reporting greater trust/hope about getting vaccinated (0–5 scale) were more likely to adhere to the vaccination campaign (OR = 1.76, CI [1.10, 2.82], *p* = .019). Conversely, individuals who experienced greater anxiety/worry at the onset of the pandemic (0–5 scale) had lower odds of vaccination adherence (OR = 0.46, CI [0.27, 0.77], *p* = .003). The remaining factors did not reach statistical significance. The complete regression results are reported in the Supplementary Material (Table S1). Bootstrap resampling (1,000 iterations) confirmed the robustness of the logistic regression model, with consistent effect directions and significant predictors. However, wider confidence intervals reflected the limited number of vaccinated participants (*N* = 35) and potential variability in estimates.

#### Risk perception

The ordinal logistic regression on the *perceived likelihood of contracting COVID-19* revealed a significant model (χ²(27) = 61.26, *p* < .0001; Nagelkerke’s R² = 0.24). Compared with participants aged ≥ 60 years, those aged 40–49 (OR = 3.42, CI [1.30, 9.01], *p* = .013) and those aged 50–59 (OR = 3.46, CI [1.58, 7.61], *p* = .002) were more likely to perceive a greater likelihood of contracting COVID-19. Participants with no previous COVID-19 infection had lower odds of perceiving a greater likelihood of infection compared with those with prior infection (OR = 0.31, CI [0.17, 0.58], *p* < .001). The participants who were not aware of current COVID-19 workplace regulations also had lower odds of perceiving a greater likelihood of infection than those who were aware (OR = 0.49, CI [0.26, 0.92], *p* = .026). Individuals reporting greater anxiety/worry at the onset of the pandemic were more likely to perceive a greater likelihood of infection (OR = 1.31, CI [1.06, 1.62], *p* = .012). The remaining factors did not reach statistical significance. The complete regression results are reported in the Supplementary Material (Table S2).

The ordinal logistic regression on the *perceived severity of the current COVID-19 spread* revealed a significant model (χ²(27) = 86.42, *p* < .0001; Nagelkerke’s R² = 0.33). Compared with participants aged ≥ 60 years, those aged 18–29 were less likely to perceive the current COVID-19 situation as more severe (OR = 0.15, CI [0.04, 0.63], *p* = .010). The participants who found access to COVID-19 information very easy were less likely to perceive greater severity than those who found access very difficult (OR = 0.11, CI [0.02, 0.51], *p* = .005). A similar but marginally significant effect emerged for participants who reported access to information as easy (OR = 0.23, CI [0.05, 1.00], *p* = .050). In contrast, participants who reported that COVID-19 information was disseminated often through mass media (compared with very often) were more likely to perceive the current situation as more severe (OR = 11.47, CI [1.57, 83.96], *p* = .016). A higher number of vaccine doses received was associated with greater perceived severity (OR = 1.58, CI [1.08, 2.32], *p* = .020). Individuals reporting greater anxiety/worry about contracting COVID-19, both currently (OR = 1.49, CI [1.16, 1.92], *p* = .002) and at the onset of the pandemic (OR = 1.40, CI [1.12, 1.74], *p* = .003), were also more likely to perceive greater severity. The remaining factors did not reach statistical significance. The complete regression results are reported in the Supplementary Material (Table S3).

#### Intention to take an antigen test in case of symptoms

The binary logistic regression on the intention to take an antigen test revealed a significant model (χ²(17) = 92.23, *p* < .0001; Nagelkerke’s R² = 0.48). Individuals who currently adopted a higher number of preventive measures (ranging from 0 to 7) had greater odds of taking the test in case of symptoms (OR = 1.56, CI [1.19, 2.05], *p* = .001). Participants with a greater number of previous vaccine doses (0–6) were also more likely to take the test (OR = 2.24, CI [1.22, 4.11], *p* = .009). Individuals experiencing greater trust/hope about the test had higher odds of taking it (OR = 2.18, CI [1.46, 3.27], *p* < .001). In contrast, individuals who experienced greater anxiety/worry about the test were less likely to take it (OR = 0.64, CI [0.43, 0.94], *p* = .023). The participants who experienced greater current anxiety/worry about contracting COVID-19 were more likely to take the test (OR = 1.71, CI [1.11, 2.64], *p* = .015). Finally, greater anxiety/worry about contracting COVID-19 at the onset of the pandemic was associated with lower odds of taking the test (OR = 0.69, CI [0.49, 0.98], *p* = .038). The remaining factors did not reach statistical significance. The complete regression results are reported in the Supplementary Material (Table S4).

Owing to the limited number of participants who did not intend to take the test (*N* = 50), a bootstrap resampling (1,000 iterations) was conducted. The analysis confirmed the stability of the estimates and improved precision, as indicated by narrower confidence intervals. However, the effect of anxiety/worry about contracting COVID-19 at the onset of the pandemic lost statistical significance (*p* = .09) and will not be further discussed.

## Discussion

Overall, four main findings emerged from the present study. First, only 13.1% of participants adhered to the 2023–2024 COVID-19 vaccination campaign, with only 30.6% of individuals aged 60 years or older, for whom vaccination was explicitly recommended [7]. Second, vaccination adherence was driven primarily by older age, a higher number of previous COVID-19 vaccine doses, and greater trust/hope related to vaccination, whereas greater anxiety/worry about contracting COVID-19 at the onset of the pandemic was associated with lower adherence. Third, risk perception concerning the likelihood of infection was predicted by being middle-aged, having previously contracted COVID-19, and being aware of workplace regulations, with anxiety/worry related to the early pandemic consistently associated with higher perceived infection risk and greater perceived severity of the current COVID-19 spread. However, risk perception did not predict vaccination adherence or test-taking intentions. Fourth, the intention to undergo antigen testing was primarily predicted by adopting a higher number of preventive measures, having received more previous COVID-19 vaccine doses, and experiencing greater trust/hope related to testing, whereas higher test-related anxiety/worry was associated with lower intention.

Our data support ECDC monitoring [6] in highlighting the failure of the 2023–2024 COVID-19 vaccination campaign in Italy. Notably, vaccination was almost absent among individuals under 40 years of age (~ 94%), consistent with previous observations [36]. While not at high risk of severe outcomes, younger populations have been identified as having a significant impact on virus transmission, thereby increasing the risk for the most vulnerable individuals [37].

Not perceiving COVID-19 as a serious health threat and feeling sufficiently protected due to prior infection were the most reported reasons for not getting vaccinated. Concerns about vaccine safety, efficacy, or potential side effects also emerged. Previous reports indicate that the factors affecting vaccine intention and uptake vary considerably across time and countries [38]. A systematic review of studies conducted between 2020 and 2022 highlighted fear of adverse reactions and doubts about vaccine efficacy as the most common determinants of vaccine hesitancy in the Italian population, besides sociodemographic factors [39]. Previous studies conducted in Italy identified low perceived risk of COVID-19 as an important factor influencing vaccine hesitancy [19, 26, 40]. However, in the present study, trust in vaccine safety and risk perception (i.e., perceived likelihood of contracting COVID-19 and perceived severity of the current spread) did not prove to be significant predictors of adherence to vaccination, nor did awareness of campaign recommendations. This pattern can be interpreted in light of both methodological and contextual considerations. First, unlike most previous studies, the present investigation focused on actual vaccination behavior during an ongoing campaign, rather than on vaccination intention or attitudes. Second, data were collected in a post-emergency phase characterized by a lower perceived threat and widespread personal experience with COVID-19, including prior infection and previous vaccination doses, which may reduce the salience of previous risk-based beliefs and safety concerns. Third, the cumulative emotional burden associated with the early pandemic may have contributed to a form of pandemic fatigue that weakens the influence of traditional cognitive predictors. Taken together, these elements may therefore account for discrepancies with previous findings. Consistent with this interpretation, vaccination history, in terms of the number of doses received, emerged as the strongest predictor, indicating that vaccination behavior was driven primarily by past compliance. Age also showed the expected pattern, with older individuals being more likely to adhere to the campaign, as consistently reported in previous research [26, 39, 40]. Importantly, affective states also emerged as significant predictors of vaccination behavior. Greater trust/hope at the thought of getting vaccinated increased adherence, whereas greater anxiety/worry about contracting COVID-19 at the onset of the pandemic had the opposite effect. Interestingly, anxiety/worry about contracting COVID-19 in the current period did not have the same influence, possibly because affective responses have markedly declined in the post-emergency phase and may coexist with conflicting feelings (see below). These findings indicate that positive emotional reactions toward vaccination may exert a more substantial influence on actual behavior than general beliefs about its safety, at least in the post-emergency pandemic phase. This interpretation is consistent with evidence showing that hope is a predominant motivating affective state among vaccination-inclined individuals [41] and a key driver of self-protective behavior [42]. While previous studies reported that current negative affective states (fear, anxiety, or worry) during the pandemic were associated with greater adherence to preventive measures and vaccination [43–45], our data suggest that the anxiety and worry experienced in the early pandemic may now act as deterrents. Initial distress may indeed have facilitated compliance when the perceived threat was high, yet prolonged exposure to negative affect could have contributed to pandemic fatigue, ultimately reducing engagement in preventive behaviors, as highlighted by a recent longitudinal study [46].

With respect to risk perception, our findings suggest that the two indicators considered in this study captured partly different underlying processes. The perceived likelihood of infection was greater among individuals with prior infection and among those reporting higher early-pandemic anxiety/worry, suggesting that risk appraisals remained shaped by personal exposure and by affective states referring to the onset of the pandemic. In contrast, the perceived severity of the current disease spread was more closely associated with access to COVID-19 information, vaccination history, and both early-pandemic and present anxiety/worry, indicating that contextual and informational cues, together with affective states, contributed to how the current epidemiological situation was evaluated. Importantly, for both indicators, anxiety-related states referring to COVID-19 emerged as significant predictors (*risk as feeling*), as previously reported in the literature [47]. Beyond the contribution of affective states, these patterns suggest that informational and experiential elements also shaped risk perception in the post-emergency phase. Difficulty in accessing information may have increased the uncertainty and perceived severity of the current spread, consistent with evidence showing that information seeking reduces uncertainty [48]. Prior infection may have heightened perceived vulnerability and awareness of the contextual factors that facilitate contagion [49, 50]. Age differences indicate that subjective risk was not aligned with actual epidemiological vulnerability, as older individuals did not report higher risk perception despite their objectively higher clinical risk, a pattern already observed in Italy during the initial stages of the pandemic [49, 50]. This broader mismatch between perceived and objective risk may help explain why risk perception no longer translated into greater adherence to vaccination and why vaccination history emerged as a strong indicator of both perceived severity and adherence to recommended preventive actions, including vaccination and testing.

Vaccination history also significantly predicted the intention to take an antigen test in case of symptoms, suggesting a consistent pattern of compliance across different preventive recommendations. The intention to test was further predicted by the number of preventive measures adopted (e.g., social distancing, handwashing, avoiding crowded places), indicating a general consistency in the adoption of behaviors aimed at limiting viral transmission. As previously documented, preventing the spread to others represented the primary motivation for testing [51]. Affective states again played a central role. Greater trust/hope about testing and greater current anxiety/worry about contracting COVID-19 increased intentions to test, whereas higher anxiety/worry about taking the test had the opposite effect. This pattern is consistent with prior evidence showing that anxiety about the disease motivates preventive actions [51–53], while highlighting that anxiety specifically tied to the testing procedure may instead deter engagement.

A novel contribution of the present study lies in the broad assessment of pandemic-related affective states, extending previous work predominantly focused on negative emotions, particularly fear and anxiety [44, 52, 53]. By examining a wide range of positive and negative emotional states, as well as feelings of control and disengagement, our findings provide a more comprehensive picture of how individuals related to COVID-19. As expected, emotional intensity markedly declined when comparing the initial pandemic phase (retrospectively rated) with the current period, consistent with previous evidence [52, 54], except for calm/tranquility and indifference, which increased. Early-pandemic feelings were dominated by fear, anxiety/worry, stress, uncertainty, and helplessness, reflecting a profound sense of insecurity. In the current phase, fear, anxiety/worry, stress, uncertainty, and helplessness coexisted with equally intense calm/tranquility and indifference toward COVID-19, indicating the presence of conflicting affective states. In contrast, the thought of undergoing vaccination and taking an antigen test predominantly elicited calm/tranquility and trust/hope. As discussed earlier, despite their relatively low intensity, affective states related to the current pandemic phase have proven to be significant predictors of risk perception, vaccination behavior, and antigen test intentions. Importantly, emotions referring to the onset of the pandemic also continued to influence current decisions. Overall, these findings extend prior evidence on the role of emotional processes in vaccination decisions [44, 45] or, more broadly, in adopting preventive behaviors [43, 55], with emerging research highlighting hope as a key motivator linked to coping and self-efficacy [42, 56].

### Limitations

Some limitations of this study should be acknowledged. First, the cross-sectional design of the study does not allow causal inferences about the observed associations, which should therefore be interpreted within a correlational framework. Future longitudinal or experimental studies are needed to clarify their temporal and causal dynamics. Second, the characteristics of the sample necessarily constrain generalizability. The study relied on a single academic population with a relatively low response rate (267/1,110), which may have produced recruitment bias and overrepresented individuals with greater engagement in COVID-19-related issues. Moreover, individuals over 70 years of age were not included because they are no longer part of the university workforce in Italy and therefore do not share the homogeneous institutional exposure that motivated the sampling strategy; as a result, the findings cannot be extended to the oldest age groups, who represent a population at particularly high risk of severe outcomes. Third, the limited number of participants who adhered to the vaccination campaign reduced the statistical power of the regression analyses and may have affected the stability of some estimates. Finally, the regression models did not rely on a fully uniform set of predictors, as covariates were selected on the basis of outcome-specific theoretical relevance and model parsimony, which necessarily limited full comparability across models.

## Conclusions

The present exploratory study advances current knowledge on COVID-19 decision-making in the post-emergency phase by clarifying the specific roles of affective states, informational exposure, and prior preventive behavior in shaping vaccination uptake, risk perception, and testing intentions. The findings indicate that risk perception no longer translates into vaccination or testing, whereas vaccination history and a consistent pattern of preventive practices reliably predict compliance with recommended actions. A key contribution lies in demonstrating both the enduring influence of early-pandemic anxiety and the stronger motivational role of positive affective responses, specifically trust and hope, relative to cognitive risk appraisals. Overall, the results illustrate how preventive behavior is reorganized in a context of reduced perceived threat and evolving institutional communication.

### Cues to action

From a public health perspective, our findings suggest specific cues to action for future informational campaigns on seasonal COVID-19 vaccination and symptom-based antigen testing: (a) emphasize hope- and trust-based messages: positive affective cues were stronger motivators than risk appraisals or safety beliefs, indicating that communication should prioritize reassurance over threat; (b) use fear appeal cautiously: while current COVID-19 anxiety may increase willingness to test when symptomatic, reactivating early-pandemic anxiety may undermine vaccination adherence; (c) highlight continuity with past protective actions among previously compliant individuals: those with a history of vaccination or preventive behaviors were the most likely to maintain adherence; (d) target disengaged age groups, particularly younger and middle-aged adults, who showed the lowest adherence but play a substantial role in transmission.

## Supplementary Information

Supplementary Material 1: Appendix A.

Supplementary Material 2: Table S1.

Supplementary Material 3: Table S2.

Supplementary Material 4: Table S3.

Supplementary Material 5: Table S4.

## Data Availability

The data supporting this study’s findings are available from the corresponding author upon reasonable request.

## Abbreviations

ANOVA: Analysis of Variance
CI: Confidence Interval
COVID-19: Coronavirus Disease 2019
ECDC: European Centre for Disease Prevention and Control
G–G: Greenhouse–Geisser
HSD: Honestly Significant Difference
OR: Odds Ratio
SPSS: Statistical Package for the Social Sciences
WHO: World Health Organization

## References

[1] World Health Organization. WHO Director-General’s opening remarks at the media briefing – 5. 2023. https://www.who.int/director-general/speeches/detail/who-director-general-s-opening-remarks-at-the-media-briefing---5-may-2023. Accessed 2 Jan 2025.

[2] Bhurani N, Umate R, Umate S, Long. COVID-19 Syndrome and its Effects on Various Systems: A Narrative Review. J Clin Diagn Res. 2023;17:LE01–5. 10.7860/JCDR/2023/64869.18311.

[3] Wiemken TL, Khan F, Puzniak L, Yang W, Simmering J, Polgreen P, et al. Seasonal trends in COVID-19 cases, hospitalizations, and mortality in the United States and Europe. Sci Rep. 2023;13:3886. 10.1038/s41598-023-31057-1. 36890264 10.1038/s41598-023-31057-1 PMC9994397

[4] Lee GYL, Lim RBT. Are self-test kits still relevant post COVID-19 pandemic? Qualitative study on working adults’ perceptions. Infect Dis Health. 2024;29:73–80. 10.1016/j.idh.2023.11.001. 38049368 10.1016/j.idh.2023.11.001

[5] Italian Ministry of Health. COVID-19 Open Data Dashboard. https://opendatamds.maps.arcgis.com/apps/dashboards/0f1c9a02467b45a7b4ca12d8ba296596. Accessed 2 Jan 2025.

[6] European Centre for Disease Prevention and Control. COVID-19 vaccination coverage in the EU/EEA during the 2023–24 season campaigns. Stockholm: ECDC. 2024. https://www.ecdc.europa.eu/sites/default/files/documents/covid19-vaccination-coverage-2023-2024.pdf. Accessed 2 Jan 2025.

[7] Italian Ministry of Health. Vaccination Legislation Archive. https://www.salute.gov.it/portale/vaccinazioni/archivioNormativaVaccinazioni.jsp. Accessed 2 Jan 2025.

[8] Ford JL, Douglas M, Barrett AK. The role of pandemic fatigue in seeking and avoiding information on COVID-19 among young adults. Health Commun. 2023;38:2336–49. 10.1080/10410236.2022.2069211. 35514105 10.1080/10410236.2022.2069211

[9] Stamm TA, Partheymüller J, Mosor E, Ritschl V, Kritzinger S, Alunno A, et al. Determinants of COVID-19 vaccine fatigue. Nat Med. 2023;29:1164–71. 10.1038/s41591-023-02282-y. 36973410 10.1038/s41591-023-02282-y PMC10202806

[10] Rashid MRA, Suhaimi ASA, Mohamad SNS, Tajjudin AIA, Roslan N, Jaffar A, et al. COVID-19 Pandemic Fatigue: A Scoping Review of the Literature. Malays J Med Health Sci. 2024;20:330–42. 10.47836/mjmhs.20.1.41.

[11] Naveed Siddiqui A, Musharaf I, Gulumbe BH. The JN.1 variant of COVID-19: immune evasion, transmissibility, and implications for global health. Ther Adv Infect Dis. 2025;12:1–13. 10.1177/20499361251314763. 10.1177/20499361251314763 PMC11783492 39896217

[12] World Health Organization. Pandemic fatigue: reinvigorating the public to prevent COVID-19. Policy framework for supporting pandemic prevention and management. Copenhagen: WHO Regional Office for Europe. 2020. https://apps.who.int/iris/bitstream/handle/10665/335820/WHO-EURO-2020-1160-40906-55390-eng.pdf. Accessed 2 Jan 2025.

[13] Michie S, West R, Harvey N. The concept of fatigue in tackling covid-19. BMJ. 2020;371:m4171. 10.1136/bmj.m4171. 33139254 10.1136/bmj.m4171

[14] Lilleholt L, Zettler I, Betsch C, Böhm R. Development and validation of the pandemic fatigue scale. Nat Commun. 2023;14:6352. 10.1038/s41467-023-42063-2. 37816702 10.1038/s41467-023-42063-2 PMC10564944

[15] Wiedicke A, Stehr P, Rossmann C. Portrayal of risk information and its impact on audiences’ risk perception during the Covid-19 pandemic: A multi‐method approach. Risk Anal. 2024;1–13. 10.1111/risa.17681. 10.1111/risa.17681 PMC12369294 39568307

[16] Slovic P, Finucane ML, Peters E, MacGregor DG. Risk as Analysis and Risk as Feelings: Some Thoughts about Affect, Reason, Risk, and Rationality. Risk Anal. 2004;24:311–22. 10.1111/j.0272-4332.2004.00433.x. 15078302 10.1111/j.0272-4332.2004.00433.x

[17] Finucane ML, Alhakami A, Slovic P, Johnson SM. The affect heuristic in judgments of risks and benefits. J Behav Decis Mak. 2000;13:1–17. https://onlinelibrary.wiley.com/doi/10.1002/%28SICI%291099-0771%28200001/03%2913%3A1%3C1%3A%3AAID-BDM333%3E3.0.CO%3B2-S

[18] Malik AA, McFadden SM, Elharake J, Omer SB. Determinants of COVID-19 vaccine acceptance in the US. EClinicalMedicine. 2020;26:100495. 10.1016/j.eclinm.2020.100495. 32838242 10.1016/j.eclinm.2020.100495 PMC7423333

[19] Caserotti M, Girardi P, Rubaltelli E, Tasso A, Lotto L, Gavaruzzi T. Associations of COVID-19 risk perception with vaccine hesitancy over time for Italian residents. Soc Sci Med. 2021;272:113688. 10.1016/j.socscimed.2021.113688. 33485215 10.1016/j.socscimed.2021.113688 PMC7788320

[20] Ferrer RA, Klein WM, Avishai A, Jones K, Villegas M, Sheeran P. When does risk perception predict protection motivation for health threats? A person-by-situation analysis. PLoS ONE. 2018;13:e0191994. 10.1371/journal.pone.0191994. 29494705 10.1371/journal.pone.0191994 PMC5832213

[21] Wise T, Zbozinek TD, Michelini G, Hagan CC, Mobbs D. Changes in risk perception and self-reported protective behaviour during the first week of the COVID-19 pandemic in the United States. R Soc Open Sci. 2020;7:200742. 10.1098/rsos.200742. 33047037 10.1098/rsos.200742 PMC7540790

[22] Kricorian K, Civen R, Equils O. COVID-19 vaccine hesitancy: misinformation and perceptions of vaccine safety. Hum Vaccines Immunother. 2022;18:1950504. 10.1080/21645515.2021.1950504. 10.1080/21645515.2021.1950504 PMC8920251 34325612

[23] Marini M, Demichelis A, Menicagli D, Mancini G, Panizza F, Bilancini E, et al. I want to be safe: understanding the main drivers behind vaccination choice throughout the pandemic. BMC Public Health. 2024;24:1111. 10.1186/s12889-024-18511-z. 38649925 10.1186/s12889-024-18511-z PMC11036553

[24] Hubert P, Hadi SA, Mojzisch A, Häusser JA. The effects of organizational climate on adherence to guidelines for COVID-19 prevention. Soc Sci Med. 2022;292:114622. 10.1016/j.socscimed.2021.114622. 34871853 10.1016/j.socscimed.2021.114622 PMC8629794

[25] Russell JA. Core affect and the psychological construction of emotion. Psychol Rev. 2003;110:145–72. 10.1037/0033-295X.110.1.145. 12529060 10.1037/0033-295x.110.1.145

[26] Caserotti M, Girardi P, Sellaro R, Rubaltelli E, Tasso A, Lotto L, et al. To vaccinate or not to vaccinate? The interplay between pro-and against-vaccination reasons. BMC Public Health. 2023;23:2207. 10.1186/s12889-023-17112-6. 37946143 10.1186/s12889-023-17112-6 PMC10634164

[27] Perrone C, Fiabane E, Maffoni M, Pierobon A, Setti I, Sommovigo V, et al. Vaccination hesitancy: To be vaccinated, or not to be vaccinated, that is the question in the era of COVID-19. Public Health Nurs. 2023;40:90–6. 10.1111/phn.13134. 36168152 10.1111/phn.13134 PMC9538072

[28] World Health Organization. Survey tool and guidance: rapid, simple, flexible behavioural insights on COVID-19. 2020. WHO/EURO: 2020-696-40431-54222. Accessed 20 Dec 2023.

[29] Ekman P. An argument for basic emotions. Cogn Emot. 1992;6:169–200. 10.1080/02699939208411068.

[30] Biggs A, Brough P, Drummond S. Lazarus and Folkman’s psychological stress and coping theory. In: Cooper CL, Quick JC, editors. The handbook of stress and health: A guide to research and practice. 1st ed. Wiley; 2017. pp. 349–64. 10.1002/9781118993811.ch21.

[31] Baratta MV, Seligman ME, Maier SF. From helplessness to controllability: toward a neuroscience of resilience. Front Psychiatry. 2023;14:1170417. 10.3389/fpsyt.2023.1170417. 37229393 10.3389/fpsyt.2023.1170417 PMC10205144

[32] Tangney JP, Stuewig J, Mashek DJ. Moral emotions and moral behavior. Annu Rev Psychol. 2007;58:345–72. 10.1146/annurev.psych.56.091103.070145. 16953797 10.1146/annurev.psych.56.091103.070145 PMC3083636

[33] Fredrickson BL. The role of positive emotions in positive psychology: The broaden-and-build theory of positive emotions. Am Psychol. 2001;56:218–26. 10.1037/0003-066X.56.3.218. 11315248 10.1037//0003-066x.56.3.218 PMC3122271

[34] McClelland RT. A plea for indifference. J Mind Behav. 2020;41:211–46. https://www.jstor.org/stable/27116208.

[35] Vittinghoff E, McCulloch CE. Relaxing the rule of ten events per variable in logistic and Cox regression. Am J Epidemiol. 2007;165:710–8. 10.1093/aje/kwk052. 17182981 10.1093/aje/kwk052

[36] Zona S, Partesotti S, Bergomi A, Rosafio C, Antodaro F, Esposito S. Anti-COVID vaccination for adolescents: a survey on determinants of vaccine parental hesitancy. Vaccines. 2021;9:1309. 10.3390/vaccines9111309. 34835239 10.3390/vaccines9111309 PMC8618373

[37] Jin Y, Hou C, Li Y, Zheng K, Wang C. mRNA vaccine: How to meet the challenge of SARS-CoV-2. Front Immunol. 2022;12:821538. 10.3389/fimmu.2021.821538. 35126377 10.3389/fimmu.2021.821538 PMC8813741

[38] MacDonald NE. Vaccine hesitancy: Definition, scope and determinants. Vaccine. 2015;33:4161–4. 10.1016/j.vaccine.2015.04.036. 25896383 10.1016/j.vaccine.2015.04.036

[39] Ferrara M, Bertozzi G, Volonnino G, Di Fazio A, Di Fazio N, Arcangeli M, et al. Learning from the Past to Improve the Future—Vaccine Hesitancy Determinants in the Italian Population: A Systematic Review. Vaccines. 2023;11:630. 10.3390/vaccines11030630. 36992216 10.3390/vaccines11030630 PMC10058125

[40] Zarbo C, Candini V, Ferrari C, d’Addazio M, Calamandrei G, Starace F, et al. COVID-19 vaccine hesitancy in Italy: predictors of acceptance, fence sitting and refusal of the COVID-19 vaccination. Front Public Health. 2022;10:873098. 10.3389/fpubh.2022.873098. 35570888 10.3389/fpubh.2022.873098 PMC9098927

[41] Chen NTN, Kee K, Villalobos BT, Ortiz M, Lee H. Do cognition and emotion matter? A study of COVID-19 vaccination decision-making in college students. Health Psychol Open. 2023;10:1–12. 10.1177/20551029231179163. 10.1177/20551029231179163 PMC10227488 37261310

[42] Sand G, Bristle J. Motivating Protective Behavior against COVID-19: Fear Versus Hope. J Aging Health. 2024;36:350–66. 10.1177/08982643221089427. 35713288 10.1177/08982643221089427 PMC9207583

[43] Harper CA, Satchell LP, Fido D, Latzman RD. Functional Fear Predicts Public Health Compliance in the COVID-19 Pandemic. Int J Ment Health Addict. 2021;19:1875–88. 10.1007/s11469-020-00281-5. 32346359 10.1007/s11469-020-00281-5 PMC7185265

[44] Bendau A, Plag J, Petzold MB, Ströhle A. COVID-19 vaccine hesitancy and related fears and anxiety. Int Immunopharmacol. 2021;97:107724. 10.1016/j.intimp.2021.107724. 33951558 10.1016/j.intimp.2021.107724 PMC8078903

[45] Fisher KA, Nguyen N, Fouayzi H, Crawford S, Singh S, Dong M, et al. From COVID-19 Vaccine Hesitancy to Vaccine Acceptance: Results of a Longitudinal Survey. Public Health Rep. 2023;138:681–90. 10.1177/00333549231176006. 37243439 10.1177/00333549231176006 PMC10235915

[46] Morris KJ, Ashida S, Ramirez MR, Tarr GA. Psychological distress as a driver of early COVID-19 pandemic fatigue: a longitudinal analysis of the time-varying relationship between distress and physical distancing adherence among families with children and older adults. BMJ Public Health. 2024;2:e001256. 10.1136/bmjph-2024-001256. 40018625 10.1136/bmjph-2024-001256 PMC11816714

[47] Savadori L, Lauriola M. Risk perception and protective behaviors during the rise of the COVID-19 outbreak in Italy. Front Psychol. 2021;11:577331. 10.3389/fpsyg.2020.577331. 33519593 10.3389/fpsyg.2020.577331 PMC7838090

[48] Bradac JJ. Theory comparison: Uncertainty reduction, problematic integration, uncertainty management, and other curious constructs. J Commun. 2001;51:456–76. 10.1111/j.1460-2466.2001.tb02891.x.

[49] Dryhurst S, Schneider CR, Kerr J, Freeman AL, Recchia G, Van Der Bles AM, et al. Risk perceptions of COVID-19 around the world. J Risk Res. 2020;23:994–1006. 10.1080/13669877.2020.1758193.

[50] Martelletti CP, Santirocchi A, Spataro P, Rossi-Arnaud C, Löfstedt RE, Cestari V. Predictors of COVID-19 risk perception, worry and anxiety in Italy at the end of the 2020 national lockdown. J Risk Res. 2022;25:1306–20. 10.1080/13669877.2022.2038245.

[51] Bevan I, Stage Baxter M, Stagg HR, Street A. Knowledge, attitudes, and behavior related to COVID-19 testing: a rapid scoping review. Diagnostics. 2021;11:1685. 10.3390/diagnostics11091685. 34574026 10.3390/diagnostics11091685 PMC8472251

[52] Wang Z, Luo S, Xu J, Wang Y, Yun H, Zhao Z, et al. Well-Being Reduces COVID-19 Anxiety: A Three-Wave Longitudinal Study in China. J Happiness Stud. 2021;22:3593–610. 10.1007/s10902-021-00385-2. 33814971 10.1007/s10902-021-00385-2 PMC7997794

[53] Ferrucci R, Averna A, Marino D, Reitano MR, Ruggiero F, Mameli F, et al. Psychological impact during the first outbreak of COVID-19 in Italy. Front Psychiatry. 2020;11:559266. 10.3389/fpsyt.2020.559266. 33240119 10.3389/fpsyt.2020.559266 PMC7667038

[54] Li Y, Luan S, Li Y, Hertwig R. Changing emotions in the COVID-19 pandemic: A four-wave longitudinal study in the United States and China. Soc Sci Med. 2021;285:114222. 10.1016/j.socscimed.2021.114222. 34418734 10.1016/j.socscimed.2021.114222 PMC8529947

[55] Kolotylo-Kulkarni M, Marakas GM, Xia W. Understanding protective behavior and vaccination adoption among US individuals during the COVID-19 pandemic: A four-wave longitudinal study. J Bus Res. 2024;179:114649. 10.1016/j.jbusres.2024.114649.

[56] Petersen MB, Christiansen LE, Bor A, Lindholt MF, Jørgensen F, Adler-Nissen R, et al. Communicate hope to motivate the public during the COVID-19 pandemic. Sci Rep. 2022;12:2502. 10.1038/s41598-022-06316-2. 35169174 10.1038/s41598-022-06316-2 PMC8847429

