# Supplementary material for: Emotions, preventive measures, and risk perception in post-emergency COVID-19: a cross-sectional study in Italian university staff during the seasonal vaccination campaign
Source: BMC Public Health. 2026 Mar 14;26:1315. doi: 10.1186/s12889-026-26782-x (PMC13101245; doi:10.1186/s12889-026-26782-x)
Supplement: Supplementary file 1 — Supplementary Material 1: Appendix A. [file 12889_2026_26782_MOESM1_ESM.docx]

**DEMOGRAPHIC AND HEALTH INFORMATION**

1. **Sex**

F


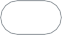


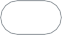
 M


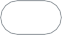
 I prefer not to answer

1. **Age**


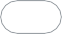
 18-29 years


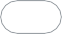
 30-39 years


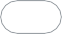
 40-49 years


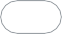
 50-59 years

Equal to or greater than 60 years


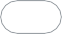


1. **What is the highest level of education you have completed?**

Primary school (elementary)


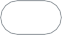


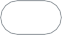
 Lower secondary school (middle school)


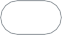
 Upper secondary school (high schools, technical institutes, vocational schools)


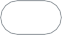
 Advanced artistic, musical, and choreographic education (academies, conservatories)


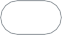
 Bachelor’s degree


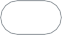
 Master’s degree (or single-cycle degree)


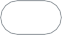
 Doctorate (PhD)

1. **What is your housing situation?**

I live alone


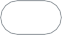


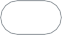
 I live with at least one person aged 60 years or older


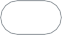
 I live with at least one person with chronic illnesses or severe vulnerabilities


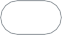
 None of the above

1. **How would you rate your health status?**

Poor


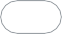


Fair


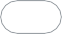


Good


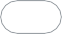


Excellent


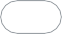


1. **Do you suffer from chronic illnesses**

Yes


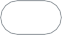


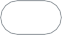
 No

**PRIOR EXPERIENCE WITH COVID-19**

1. **Have you ever contracted COVID-19?**


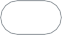
 Yes


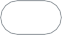
 No

If yes…

1. **In what form?**

Asymptomatic


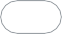


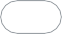
 Mild

Moderate


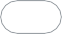


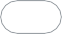
 Severe

1. **Has any of your family members or close relatives ever contracted COVID-19?**


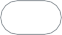
 Yes


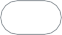
 No

If yes…

1. **Considering the person who experienced the most severe form of COVID-19 among your family members or close relatives, what was the severity of their illness?**

Asymptomatic


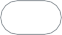


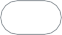
 Mild

Moderate


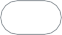


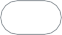
 Severe


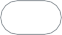
 Passed away due to COVID-19

**ACCESS TO COVID-19 INFORMATION**

1. **How easy/difficult is it currently to obtain information about COVID-19 (e.g., spread, symptoms, treatments, recommendations)?**


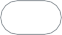
 Very easy


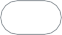
 Easy


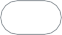
 Difficult


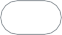
 Very difficult

1. **How often is information about COVID-19 currently disseminated through mass media?**


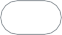
 Rarely


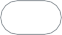
 Occasionally


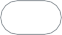
 Often


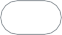
 Very often

1. **How easy/difficult is it currently to obtain information on what to do if you think you have contracted COVID-19?**


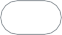
 Very easy


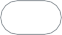
 Easy


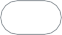
 Difficult


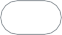
 Very difficult

1. **Are you aware of the current regulations regarding the procedure to follow in the workplace in case of a positive COVID-19 test?**

Yes


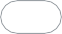


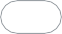
 No

**TRUST IN SOURCES OF COVID-19 INFORMATION**

1. **How much trust do you currently place in the following sources of information about COVID-19?**

|  | None | Little | Moderate | Much | Complete |
| --- | --- | --- | --- | --- | --- |
| Television |  |  |  |  |  |
| Newspapers |  |  |  |  |  |
| Healthcare professionals |  |  |  |  |  |
| Social media |  |  |  |  |  |
| Radio |  |  |  |  |  |
| Ministry of Health |  |  |  |  |  |
| National Institute of Heath |  |  |  |  |  |
| World Health Organization |  |  |  |  |  |

# RISK PERCEPTION

1. **How likely do you currently think it is that you might contract COVID-19?**


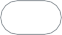
 Unlikely


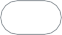
 Fairly likely


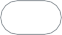
 Very likely

1. **If you were to contract COVID-19, what severity do you think would be most likely at present?**


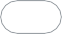
 Asymptomatic


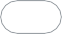
 Mild


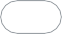
 Moderate


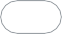
 Severe

1. **How severe do you consider the current situation regarding the spread of COVID-19 in Italy?**


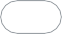
 Not severe at all


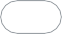
 Slightly severe


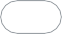
 Moderately severe


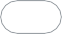
 Very severe

**USE OF ANTIGEN TESTS**

1. **If you experienced symptoms that might be attributable to COVID-19, would you take a rapid antigen test (nasal swab)?**


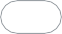
 Yes


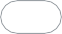
 No

If Yes…

1. **What is the *main* reason you would take the test?**


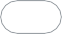
 To receive appropriate care in case of a positive result


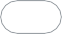
 To avoid transmitting the virus to others


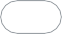
 To help limit the global spread of the virus

To gain greater awareness and control over my health


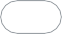


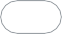
 To protect vulnerable/elderly family members or household members


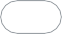
 Other (please, specify)

If Not…

1. **What is the main reason you would not take the test?**


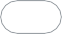
 Because it would be time-consuming and costly


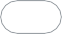
 Because I would not know where or how to take the test


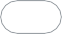
 Because a positive result might lead to restrictions in work activities and social interactions


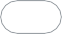
 Because I do not consider rapid tests sufficiently reliable


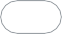
 Because even with a positive result, I would not change my behavior or habits


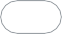
 Other (please, specify)

# VACCINATION

1. **How much trust do you have in the safety of the current COVID-19 vaccines?**


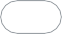
 None


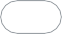
 Little


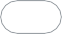
 Moderate


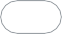
 Much


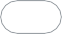
 Complete

1. **How many doses of the COVID-19 vaccine have you received so far? [Please, enter a number]**

_____

1. **Are you aware of the content of the guidelines and recommendations for the 2023-2024 autumn/winter COVID-19 vaccination campaign?**


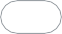
 Yes


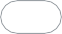
 No

1. **Have you been vaccinated as part of the 2023-2024 autumn/winter COVID-19 vaccination campaign?**


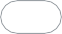
 Yes


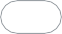
 No


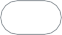
 I have made a reservation but have not yet received the dose

If Yes…

1. **What is the *main* reason you decided to get vaccinated?**


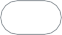
 Because I belong to one of the categories for whom vaccination is recommended, and I want greater protection against the virus and its effects


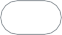
 Because I am a family/household member of a person with severe vulnerabilities or an elderly person, and I want to protect them


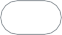
 Because, even though I do not belong to a high-risk category, I want greater protection against the virus and its effects

To help reduce the spread of the virus within the community


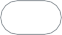


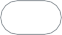
 To feel freer in social interactions and contacts

Because I was persuaded by others (e.g., family, friends, colleagues, general practitioner)


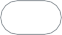


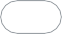
 Other (please, specify)

If Not…

1. **What is the *main* reason you decided not to get vaccinated?**


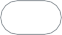
 Because I fear potential adverse effects of the COVID-19 vaccine or possible interactions with other medical conditions


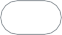
 Because I am not convinced of the efficacy or safety of the COVID-19 vaccine


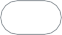
 Because I do not believe in the efficacy or safety of vaccines in general


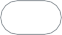
 Because I was unable to obtain the vaccine from pharmacies or my general practitioner

Because I consider myself not at risk, as many people are already vaccinated or have already contracted the disease


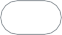


Because I currently do not see COVID-19 as a serious threat to my health


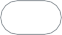


Because I believe I am sufficiently protected, having previously contracted COVID-19

Other (please, specify)

1. **If you live with vulnerable individuals or with people aged 60 years or older, have they been vaccinated as part of the 2023-2024 autumn/winter COVID-19 vaccination campaign?**

Yes

No

Not all of them

I do not live with vulnerable individuals or with people aged 60 years or older

# PREVENTIVE BEHAVIOR

1. **Which of the following measures are you currently adopting to prevent COVID-19 infection? [Select all that apply]**

I frequently wash/disinfect my hands

I avoid particularly crowded places

I wear a mask in particularly crowded places

I maintain physical distance from strangers whenever possible

I regularly disinfect surfaces

I avoid close contact with people showing symptoms similar to those of COVID-19

I avoid contact with family members who test positive for COVID-19

I do not adopt any preventive measures.

Other (please, specify)

# AFFECTIVE STATES

**Please respond spontaneously, without dwelling too much on each answer.**

1. **To what extent, on a scale from 0 to 5, does the thought of contracting COVID-19 *today* evoke the following emotions and feeling states?**

0 = None/no intensity, 5 = Maximum intensity

|  | 0 | 1 | 2 | 3 | 4 | 5 |
| --- | --- | --- | --- | --- | --- | --- |
| fear |  |  |  |  |  |  |
| anxiety/worry |  |  |  |  |  |  |
| stress |  |  |  |  |  |  |
| anger/frustration |  |  |  |  |  |  |
| sense of trust/hope |  |  |  |  |  |  |
| sense of calm/tranquility |  |  |  |  |  |  |
| sadness |  |  |  |  |  |  |
| pride |  |  |  |  |  |  |
| guilt |  |  |  |  |  |  |
| shame |  |  |  |  |  |  |
| sense of helplessness |  |  |  |  |  |  |
| sense of uncertainty |  |  |  |  |  |  |
| indifference |  |  |  |  |  |  |

1. **To what extent, on a scale from 0 to 5, did the thought of contracting COVID-19 *at the onset of the pandemic* evoke the following emotions and feeling states?**

0 = None/no intensity, 5 = Maximum intensity

|  | 0 | 1 | 2 | 3 | 4 | 5 |
| --- | --- | --- | --- | --- | --- | --- |
| fear |  |  |  |  |  |  |
| anxiety/worry |  |  |  |  |  |  |
| stress |  |  |  |  |  |  |
| anger/frustration |  |  |  |  |  |  |
| sense of trust/hope |  |  |  |  |  |  |
| sense of calm/tranquility |  |  |  |  |  |  |
| sadness |  |  |  |  |  |  |
| pride |  |  |  |  |  |  |
| guilt |  |  |  |  |  |  |
| shame |  |  |  |  |  |  |
| sense of helplessness |  |  |  |  |  |  |
| sense of uncertainty |  |  |  |  |  |  |
| indifference |  |  |  |  |  |  |

1. **To what extent, on a scale from 0 to 5, does the thought of getting vaccinated against COVID-19 *today* evoke the following emotions and feeling states?**

0 = None/no intensity, 5 = Maximum intensity

|  | 0 | 1 | 2 | 3 | 4 | 5 |
| --- | --- | --- | --- | --- | --- | --- |
| fear |  |  |  |  |  |  |
| anxiety/worry |  |  |  |  |  |  |
| stress |  |  |  |  |  |  |
| anger/frustration |  |  |  |  |  |  |
| sense of trust/hope |  |  |  |  |  |  |
| sense of calm/tranquility |  |  |  |  |  |  |
| sadness |  |  |  |  |  |  |
| pride |  |  |  |  |  |  |
| guilt |  |  |  |  |  |  |
| shame |  |  |  |  |  |  |
| sense of helplessness |  |  |  |  |  |  |
| sense of uncertainty |  |  |  |  |  |  |
| indifference |  |  |  |  |  |  |

1. **To what extent, on a scale from 0 to 5, does the thought of taking an antigen test *today* evoke the following emotions and feeling states?**

0 = None/no intensity, 5 = Maximum intensity

|  | 0 | 1 | 2 | 3 | 4 | 5 |
| --- | --- | --- | --- | --- | --- | --- |
| fear |  |  |  |  |  |  |
| anxiety/worry |  |  |  |  |  |  |
| stress |  |  |  |  |  |  |
| anger/frustration |  |  |  |  |  |  |
| sense of trust/hope |  |  |  |  |  |  |
| sense of calm/tranquility |  |  |  |  |  |  |
| sadness |  |  |  |  |  |  |
| pride |  |  |  |  |  |  |
| guilt |  |  |  |  |  |  |
| shame |  |  |  |  |  |  |
| sense of helplessness |  |  |  |  |  |  |
| sense of uncertainty |  |  |  |  |  |  |
| indifference |  |  |  |  |  |  |
